# Measuring the impact and costs of a universal group based parenting programme: protocol and implementation of a trial

**DOI:** 10.1186/1471-2458-10-364

**Published:** 2010-06-23

**Authors:** Douglas E Simkiss, Helen A Snooks, Nigel Stallard, Shan Davies, Marie A Thomas, Becky Anthony, Sarah Winstanley, Lynsey Wilson, Sarah Stewart-Brown

**Affiliations:** 1 Health Sciences Research Institute, Warwick Medical School, University of Warwick, Coventry, CV4 7AL, England; 2 Centre for Health Information Research and Evaluation, School of Medicine, Swansea University, SA2 8PP, Wales; 3 School of Health Science, Swansea University, SA2 8PP, Wales

## Abstract

**Background:**

Sub-optimal parenting is a common risk factor for a wide range of negative health, social and educational outcomes. Most parenting programmes have been developed in the USA in the context of delinquency prevention for targeted or indicated groups and the main theoretical underpinning for these programmes is behaviour management. The Family Links Nurturing Programme (FLNP) focuses on family relationships as well as behaviour management and is offered on a universal basis. As a result it may be better placed to improve health and educational outcomes. Developed in the UK voluntary sector, FLNP is popular with practitioners, has impressed policy makers throughout the UK, has been found to be effective in before/after and qualitative studies, but lacks a randomised controlled trial (RCT) evidence base.

**Methods/Design:**

A multi-centre, investigator blind, randomised controlled trial of the FLNP with a target sample of 288 south Wales families who have a child aged 2-4 yrs living in or near to Flying Start/Sure Start areas. Changes in parenting, parent child relations and parent and child wellbeing are assessed with validated measures immediately and at 6 months post intervention. Economic components include cost consequences and cost utility analyses based on parental ranking of states of quality of life. Attendance and completion rates and fidelity to the FLNP course delivery are assessed. A nested qualitative study will assess reasons for participation and non-participation and the perceived value of the programme to families. By the end of May 2010, 287 families have been recruited into the trial across four areas of south Wales. Recruitment has not met the planned timescales with barriers including professional anxiety about families entering the control arm of the trial, family concern about video and audio recording, programme facilitator concern about the recording of FLNP sessions for fidelity purposes and delays due to the new UK research governance procedures.

**Discussion:**

Whilst there are strong theoretical arguments to support universal provision of parenting programmes, few universal programmes have been subjected to randomised controlled trials. In this paper we describe a RCT protocol with quantitative and qualitative outcome measures and an economic evaluation designed to provide clear evidence with regard to effectiveness and costs. We describe challenges implementing the protocol and how we are addressing these.

**Trial Registration:**

Current Controlled Trials ISRCTN13919732

## Background

Parenting has been shown to play a part in determining future mental health (child and adolescent [1,2] and adult [1,3,4]), health related lifestyles (including healthy eating [5], substance misuse [6] and teenage pregnancy [7]), injury rates [8], aspects of physical health [3,9-12], social competence [13,14] and educational achievement [15,16]. Suboptimal parenting is therefore a risk factor for a wide range of health outcomes and improvements in parenting could contribute to the achievement of a range of current policy goals. Through its impact on educational achievement and social competence, parenting is also a determinant of future employability and thus of social inequalities in future generations. Parenting is by no means the only influence on these outcomes. Both the child's temperament [17] and their genetic makeup play a part together with environmental and social factors. However, there is some evidence to suggest that children with problem temperaments are particularly susceptible to suboptimal parenting [18] and that parenting also interacts with genetic risk [19]. So even in families with the other risk factors, parenting support may be a useful intervention. Parenting is amenable to intervention through group parenting programmes and the evidence base showing that such programmes are cost effective in treating conduct disorder and child behaviour problems is strong [20,21]. There is good evidence that they are also effective in preventing behavioural problems in high-risk groups identified by socio-economic deprivation, ethnic group and experience of life events [22-25]. Most of the evidence base on parenting programmes in the early years relates to two programmes: the Incredible Years (IY) Programme [26,27] developed in the USA and Triple P (TP) [28,29] developed in Australia, both of which are now available in the UK. Both focus on the prevention and treatment of conduct disorder, crime and delinquency [25,29] through a targeted approach.

Parenting interventions can be provided on a universal, targeted or indicated basis. The latter approaches have the strongest evidence base and lowest initial cost [20-25]. However, strong arguments can be advanced to suggest that universal provision increases the likelihood of change in high risk as well as whole population groups [30-32]. Given the range and ubiquity of health and social outcomes on which parent-child relationships have an influence [2,3,5-16], the inefficiency of targeting on the basis of identifiable risk factors [30,31], and the prevalence of sub-optimal parenting [33], universal approaches are appealing. As a result, most current government policies relating to parenting in the UK recognise the need for a universal component [34,35], ranging from provision of information and support to universal access to parenting programmes [36].

Until recently very little rigorous evaluation of effectiveness has been conducted on parenting programmes in the UK, but there has been a significant level of programme development. Several UK developed programmes, including the Family Links Parenting Programme [37] (FLNP), are now widely available. These programmes have much in common with IY and TP but focus more on the quality of the parent-child relationship and the parents' well-being. The FLNP is popular with practitioners who see it as having a distinct contribution to make to promotion of health and well-being. The current evidence base for this programme includes qualitative research showing that parents, recruited through schools, value the programme and perceive it to have an impact on family relationships, children's behaviour and their own mental health [38]; before and after studies in community groups showing impact on self report measures of relationship quality and well-being [39]; and routine evaluation by parents attending programmes showing that the great majority value the programme [40]. None of this evidence allows an estimation to be made of the extent of programme impact relative to changes occurring in a control group or enables economic modelling of the costs and effects necessary to assess the relative value of expenditure on such programmes. Such evidence is essential to inform decision making on the best approach to provision of support for parenting.

Lack of an RCT evidence base makes commissioners reluctant to fund provision of the FLNP and because of concern that the programme might no longer be provided in early years settings in their localities, practitioners and commissioners in four counties in south Wales and the Welsh Assembly Government identified funding to commission an RCT of the programme. As a result of this commissioning process which included national open competition and external, transparent peer review, the trial has been adopted as a Clinical Research Collaboration Cymru (National Institute for Health Research umbrella) portfolio study.

This trial aims

1. To measure the effectiveness of the Family Links Nurturing Programme (FLNP), in securing beneficial impact on parenting and health and social outcomes for young children and their families in the short and medium term.

2. To measure the cost consequences of the FLNP.

3. To investigate the fidelity of programme implementation and delivery by practitioners in the trial sites.

4. To investigate the views of families receiving the FLNP in the trial sites related to perceived value attributed to the programme.

## Methods/Design

The study design is a multi-centre, investigator blind, randomised controlled trial.

### a) Setting

The sample is being recruited from families of children aged 2-4 years in deprived areas of Cardiff, Newport, Torfaen and Caerphilly in south Wales. The sampling approach provides a geographical spread and coverage of different cultural groups and should recruit families representative of the UK population in relatively deprived areas, the group for whom universal programme implementation is most clearly recommended and most likely to prove cost effective.

### b) Sample size calculation

We aimed to recruit 144 families into each of the control and intervention groups (288 in total) This sample size will be sufficient to detect an effect size of 0.4 at 85% power and alpha of 0.05 in the primary outcome measure. An effect size of 0.4 is the level of difference which could be expected on the basis of changes observed on objective measures of parenting in recent UK trials of the IYs programme [25,41], in the majority of trials included in systematic reviews [21,22] and in a before and after study of FLNP [40] using self report measures. The control families are offered FLNP after data collection at six months 'post intervention' is complete.

Since the intervention is offered to groups a cluster design effect is included in the analysis. Such design effects are typically small in parenting programme trials. One recent study of the IYs programme in which both randomisation and intervention were subject to design effects identified intra-class correlations attributable to 'group' effects ranging from 0 to 0.16 [25]. In our study, potential design effects would be attributable to the group-based nature of the intervention only, as randomisation is being undertaken at the level of individual families. If the intra-class correlation in our trial is as high as 0.178, a sample of 288 will be sufficient to detect an effect size of 0.6.

Families who do not attend or dropout will be followed up alongside other families. We anticipate a 20% loss to follow-up at 6 months post-intervention based on the results of other trials [42].

### c) Recruitment

Consecutive parents visiting early years' centres are being approached by practitioners. Those who agree, meet with researchers either in the early years centre or on a home visit to discuss the research and to assess eligibility. A repeat visit is made to those still interested 1-2 weeks later for consent, collection of baseline data and randomisation (via the Warwick Clinical Trials Unit randomisation service) to allocate families to control or intervention groups. In families with more than one parent/parent figure present in the household, both parents are being invited to attend the groups, but data are being collected only from the identified main carer. Families were offered a £20 voucher for each tranche of data. In families with more than one child in the age range, data are being collected for both children. Lack of independence of data from multiple children in one family will be taken into account in the analysis (see below).

### d) Intervention

The FLNP is a ten week programme involving 2 hour sessions each week for groups of 6-10 parents. The programme is structured and aims to provide experiential learning through the use of guided discussion, role play and home work. Parents set targets for themselves each week and report back on progress the following week. The course covers a wide range of behaviours and attitudes which parents may or may not choose to adopt. The ethos of the programme is that to empower parents to bring about changes in family life it is important to invite them to test different approaches and see which they can make work in their families, rather than providing prescriptive instructions.

The four building blocks of the programme are: the development of self-awareness and self-esteem, appropriate expectations, positive discipline, and empathy. The programme is eclectic, drawing on social learning theory and psychotherapeutic insights. It is founded on the belief that empathetic insight into emotional determinants of behaviour is important for both positive relationships and behaviour management. It aims to provide parents with insights into the origins of self-esteem and positive relationships by drawing on their own experiences as children. The programme thus supports parents in improving their own relationships with others as well as with their children. Parents are given a copy of the programme book 'The Parenting Puzzle' [37] and each programme is run by two trained facilitators who receive face to face supervision three times during the course of the programme from an experienced programme facilitator. The capacity to make compassionate relationships with the parents in the groups, to empower and to support them and at the same time provide a tightly run structured group programme is seen as essential to success [26,43].

### e) Outcome measures

Parenting programmes can affect family life and wellbeing in a variety of ways and families may respond differently. No single outcome measure can capture all possible impacts. Our primary outcome measure is a composite index derived from observations made and questions administered during a home visit and recorded according to the HOME inventory [44], and responses to a parent report measure of parent child relations (adapted Mothers' Object Relations Scales (MORS), unpublished data) collected at the same visit. This measure is based on an index developed for the recent 3 year evaluation of Sure Start [45] in the UK which has provided data that are normally distributed and sensitive to change.

To supplement this primary outcome, we have included objective measures of parenting: - a 10 minute video of a mealtime coded according to the Mellow Parenting Scheme [46]. This provides scores on six dimensions of parenting; Anticipation of Child's Needs, Responsiveness, Autonomy, Cooperation, Child Distress and Control/Conflict; and a five minute speech sample capturing the parents' description of their children and their relationship with each child.

We have included a range of validated and well used secondary outcome measures to capture health and wellbeing in parents and children. For child wellbeing we have included PedsQL: parent report [47] and the pre-Parent Account of Child Symptoms (prePACS) [48], a standardised interview covering attentiveness, antisocial behaviour and emotional problems together with mother's rating of the scale of the problem and her capacity to cope. Parental wellbeing is assessed using WEMWBS [49], a 14 item self report measure of positive mental health and wellbeing, Parenting Stress Index [50] and a self report quality of life measure: SF-6D [51]. The PedsQL scale will be used as a parent proxy report of the health related quality of life of their child in order to produce utility estimates for the children in each arm of the study [47].

The objective measures of parenting (the video and speech sample) are collected during a home visit to the families in the two months before the programme starts and again six months post programme. Self-completion questionnaires are collected pre intervention, immediately post intervention, and six months post programme. Videos of parenting and five minute speech samples are coded up by researchers who do not know the families and all data analyses will be carried out blind to group allocation.

Cost consequences are being established using health, social service, educational psychology or criminal justice service contacts for children and parents, and use of voluntary sector services; parental expenditure and time away from work attributable to these events or to involvement in the FLNP. Unit costs will be attached using routine data sources [52]. The cost consequences analysis will provide a clear descriptive summary of the results for each of the outcome measures set against the cost of FLNP.

The views and perceptions of families receiving the programme are being assessed by interviews with 12 parents who decline to take part in the research study to determine possible ways of improving recruitment and 12 intervention group parents representing different cultural and social backgrounds and different experiences (positive and negative) within the group, to gather information on the most and least valued aspects of programme, rating of the facilitators, and ways of improving the programme.

Programme fidelity is being assessed using video recordings of three random sessions in each programme (with parental consent) coded by Family Links staff. In addition both the parent and facilitator evaluation forms for the recorded sessions will be reviewed by Family Links. Uptake rates; attendance rates (number of sessions attended); dropout rates; mother/father ratio of attendees and attendance and dropout by day and time of group are also being measured.

### f) Analysis

The primary statistical analysis aims to measure effectiveness in a pragmatic real life context; this will be an analysis by allocated treatment (based on all families allocated to the intervention group regardless of whether they attended the programme), using mixed univariate and multivariate analysis of variance, taking into account level of attendance, programme fidelity and differences in the characteristics of the two groups as measured by collection of data from parents at baseline: parental age, marital status, housing tenure, income, ethnicity, parental health (including mental health) and recent stressful life events. As a supplementary analysis the potential of the programme to enable parents to change will be assessed in a regression analysis based on numbers of sessions attended (efficacy analysis).

Secondary analyses will be undertaken of all other outcome measures adopting first an allocated treatment approach using mixed univariate and multivariate methods as above followed by an efficacy analysis.

Intra class correlations will be assessed for all the main outcome measures and multilevel modelling used to take into account any design effects as well as the non independence of data from multiple children in one family.

A cost consequences analysis, which allows an array of outcome measures to be considered alongside the cost of the programme will be undertaken to compare FLNP with no intervention in parents and children from public purse, societal and parents' perspectives. Modelling will examine the sensitivity of the results to a range of assumptions.

Video recordings of sessions will be analysed using an agreed structured coding framework to assess the extent to which facilitators achieved programme fidelity and the skills of the facilitator, with a simple 1-5 fidelity rating applied to each programme.

For the qualitative interviews an iterative thematic analysis will be undertaken using data from all sources independently and results 'triangulated' across these to identify areas of commonality and difference.

### g) Ethics

Ethical approval for the overall study was granted by the North West Wales Research Ethics Committee (08/Wno01/50) on 6^th ^October 2008 after the parent information leaflet and consent form were translated into Welsh. No other issues were raised by the committee. After piloting the outcome measures we adapted the measures to those outlined above and sought approval for these measures. Approval of this substantial amendment (AM01) was given by the same Research Ethics Committee on 22nd December 2008.

### h) Trial Steering Group

An independent Trial Steering Committee was established to monitor the progress of the trial including independent statisticians and trialists, a child psychiatrist, am educational psychologist and two lay/parent members and advise on modifications to the protocol as and when necessary.

In this section of the paper we report on the implementation of the trial in south Wales, including recruitment and follow up against our targets; barriers identified during this process and adaptations made by the research team in order to address these challenges; and other issues encountered in relation to the trial protocol implementation.

### i) Recruitment against targets

The original aim was to recruit 288 families to the trial at three sites, in two phases running in September 2008 and January 2009 as the FLNP runs during a school term. However contractual processes delayed the appointment of Research Officers until December 2008 and only 25 families joined the trial for the January 2009 courses. With more recruitment time, the addition of another trial site and additional support from the Primary Care Research Network at Clinical Research Collaboration Cymru (CRCC), recruitment improved to 87 families by the end of Phase 2. On the advice of the trial steering committee, third, fourth and fifth recruitment phases have been agreed to meet the trial sample size (Table 1) and trial funding extended accordingly. The trial protocol included an option to recruit from general practice if centre based recruitment proved too slow. Ethical approval was gained for this eventuality in the original ethics submission. During the fourth and fifth phase of recruitment an attempt was made to invoke this approach. This had to be aborted because research governance approval to extend the trial into general practice took eight months to arrive.

**Table 1:** Recruitment

	Phase One (January 2009)	Phase Two (April 2009)	Phase Three (September 2009)	Phase Four (January 2010)	Phase 5 (May 2010)
Caerphilly	n/a	4	5	12	17
Cardiff	11	20	28	20	11
Newport	3	25	15	35	5
Torfaen	11	13	21	21	10

Phase total **Cumulative total**	25 **25**	62 **87**	69 **156**	88 **244**	43 **287**

### j) Recruitment processes in each area

**Figure 1:**
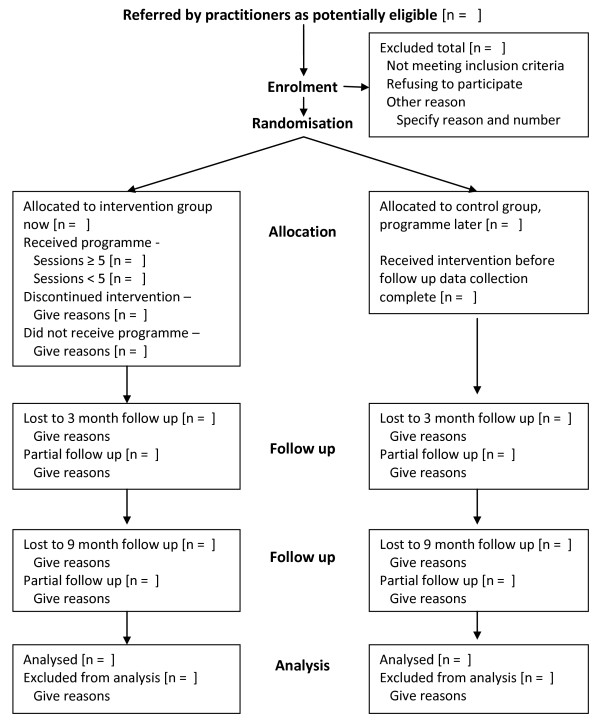
**the FLNP RCT CONSORT Flowchart**.

There were some operational differences in the recruitment process across the four sites participating in the trial, but the process is broadly as follows: a programme coordinator or administrator received a referral form or informal information about families interested in the Family Links Nurturing Programme. These referral forms come from a number of sources, for example, health visitors, social services and childcare practitioners. Each referral to the study is assessed using the trial eligibility form. Those eligible to take part are given information about the research and asked if they agree to the researcher contacting them to discuss the trial. The researcher organises a home visit to those families who agree, explains about the research and gives an information sheet. Those families who agree to take part have a second visit from the researcher to complete the baseline outcome measures. Figure 1 describes flow of participants through the trial.

### k) Adjustments made to initial planned methods of recruitment

Recruitment processes have changed over time as experience and new ideas were shared across the four sites. The more effective recruitment methods are:

1. Developing and distributing more promotional material (leaflets, posters, etc)

2. Building up relationships with practitioners at childcare settings and promoting courses before/during/after crèche

3. Developing a newsletter for practitioners

4. Attending groups (such as healthy eating, breastfeeding or clinic) where parents were already involved in centre-based activities.

5. Attending events such as play days, etc which children between the ages of 2 and 4 and their parents attend

6. Sending out letters to parents promoting FLNP course and trial alongside the offer of free childcare

7. Sending out letters for parents of children at nursery schools promoting FLNP course and trial

8. Arranging get togethers of practitioners across trial sites

### l) Issues relating to recruitment

Throughout recruitment each area presented its own challenges, but some of the barriers were common to all areas. The main problems and solutions, where achieved, are outlined below.

#### Referrals

A major barrier was professional anxiety about families entering the control arm of the trial and concern about the welfare of the family while they waited for the program. It was important that the practitioners providing referrals fully understood the rationale for the trial and its ethical approval; however, a number of problems emerged at the referrals stage:

1. Delays in referrals with no understanding of the limited recruitment 'window' for each phase of the trial

2. Incomplete information on referrals

3. Inappropriate referrals e.g. parents ineligible for the research with children too old or young or because a court order required a parenting course attendance

4. Reluctance on the part of practitioners to promote the programme

5. Professionals transmitting their own concerns over the research to potential participants e.g. by assuming the video element would cause anxiety

6. Difficult relationships between professionals and potential participants

It proved necessary to brief practitioners on more than one occasion and for the trial steering committee and chief investigator to send letters to managers outlining the consequences of failing to recruit on more than one occasion.

These issues were tackled by re-educating practitioners about the nature of the research, and in Caerphilly, recruitment was opened up to a valley outside the Flying Start area, in an effort to gain more referrals.

#### Turning referrals into participants

The participants often belong to hard to reach groups, with a high level of literacy problems which prevents the widespread use of written material; poor attendance at meetings; and difficulties in establishing and maintaining contact with potential participants by phone or at the given address.

Some potential participants could not be recruited because they set conditions on attendance, examples included: only wanting to take part in the course with a friend or relative, unwilling to do it alone, wanting to do the programme straight away and so being unwilling to risk allocation to the control group. Mistrust was another factor, families presumed that people will judge the way they parent and became defensive.

Some participants were concerned about data collection, this generally centred on the filming. Worries included being judged on cleanliness or tidiness of their home, concern that a dietician would look at what the children were eating and anxiety that Social Services or some other organisation would see the videos. Other families said that they could not be filmed because of religious or cultural issues. In order to maintain high recruitment rates, it was necessary to make the video optional.

Although time consuming, visits in person were more effective than phone calls or letters in some areas and tea and toast appeared to improve attendance at coffee mornings. However, heavy snow fall in early 2009 prevented visiting at a key recruitment time and in some instances group approaches proved counterproductive. For example a dominant parent who did not like the sound of the trial was able to 'turn off' an entire group who might have had a different response if approached individually.

#### Conducting baseline visits

Contacting people for baseline visits also raised many of the above issues. In addition some participants raised concerns that the information may impact on their receipt of benefits payments.

#### Fidelity filming

There was some facilitator anxiety about the video recording of FLNP sessions, to establish programme fidelity. These facilitators felt they might be being judged and would not facilitate any groups involved in the research. Other facilitators would not promote the trial to ensure the group they were running was not evaluated.

#### Follow up rates at date

Table 2 shows follow up rates immediately post intervention and table 3 shows follow up rates 6 months post intervention.

**Table 2:** Follow up rates immediately post intervention

	Phase One	Phase Two	Phase Three
Caerphilly	n/a	4/4	4/5
Cardiff	7/11	18/20	28/28
Newport	3/3	18/25	13/15
Torfaen	11/11	12/13	16/21

(15% drop out rate)

**Table 3:** Follow up rates 6 months post intervention

	Phase One	Phase Two
Caerphilly	-	4/4
Cardiff	8/11	18/20
Newport	3/3	19/25*
Torfaen	10/11	12/13

(15% drop out rate)

* One participant lost to follow up immediately post intervention completed the 6 month post intervention data collection

#### Reasons for loss to follow up

Follow up rates have generally been high, and have exceeded those expected. However, some families have been lost to follow up, due to the following factors:

•Some parents no longer feel the research is important at follow up

•A prolonged postal strike in autumn 2009 resulted in delayed or lost immediate post intervention questionnaire; many parents stated that they either did not receive the questionnaires or that they had already sent them back, even though they were not received by the researchers.

•Changes in address and/or phone numbers without informing anyone connected to the study.

•Changes in family dynamics/situations, particularly families involved with social services meaning that follow up is no longer possible (e.g. child taken into public care or removed to care of grandparent).

The researchers have attempted to overcome these difficulties by resorting to home visiting to collect the questionnaires and offering to help complete them when literacy problems were acting as a deterrent.

#### Trial protocol implementation: challenges to researchers remaining blind to allocation

Every care is being taken to ensure that researchers do not come into contact with trial participants allocated to the intervention arm when they gather recordings for fidelity coding (by leaving cameras before people arrive and collecting them after the end of the sessions) and this strategy has been successful. However whilst participants are clearly asked not to let the researcher know which groups they are in, some do give away whether they had been on the course or not when visited for follow-up visits; those who have been on the course and had enjoyed it may be enthusiastic to let the researcher know about the experience, and those on the waiting list may ask how much longer they would have to wait for the course.

## Discussion

Randomised controlled trials of complex interventions are challenging to carry out for a number of reasons. Most obviously participants cannot be blind to intervention and it can be difficult, as in this case, to maintain complete blinding of the research team. Even highly professional researchers can be affected by knowledge of which group participants are in and the potential for bias will need to be considered in interpreting results. Objective outcomes which can be coded blind to group allocation are important in this respect. However, in this study these have not proved unproblematic with some parents refusing to be videoed with their child at a meal time for fear that this might be used as 'evidence' against them by, for example, social services. Interventions such as parenting programs are most likely to be made available free of charge to families living in deprived circumstances, but such families are often suspicious of authority in one form or another and may not value research in this same way as other families. Recruiting and retaining such families presents particular challenges. We adopted a strategy of giving parents a gift for each tranche of data they provided and believe this has increased retention. Researchers have acted with great sensitivity and respect towards families and parents and many have said that they value the researcher visits. So far our follow up rates have remained remarkably good given the challenging circumstances.

We were not anticipating that practitioners might also present a barrier to recruitment. As this trial was funded by the County Councils because of practitioner concerns that without an evidence base the programme might no longer be funded, and because managers and practitioners attending a practitioners' group for the trial were enthusiastic and very keen to make it work, we believed initially that practitioners would all be supportive. This turned out not to be the case. Research staff had to undertake many further discussions with practitioners in groups or on a one to one basis to explain the need for randomisation and convince them that it was in their interests and those of the families to support recruitment to the trial. The existence of a practitioner group bringing together practitioners and managers across the four areas has enabled the practitioners and researchers to learn from each other's ideas and overcome many of these obstacles to recruitment.

With five phases we have almost recruited to the sample size estimation of 288, but this has taken a year longer than originally planned. The support of the Trial Steering Committee was important in persuading the County Councils that the research team were doing all that could be done to achieve the necessary level of recruitment and that additional funding was required to extend the recruitment period.

The group based nature of this programme and the fact that programmes can be run only three times a year in term time also presents challenges for recruitment. Unless a parent has been to the first session they cannot join a group, although they can of course drop out. Recruiting too early may mean that families lose interest, by the time the programme starts. This has meant that there are only three windows for recruitment each year. In the first year, one of these windows corresponded to a time in which there was an extremely heavy snow fall and the valleys of South Wales were not easily accessible.

When we designed the trial we assumed that each parenting programme would be filled with trial participants. However because of challenges to recruitment and because groups are not viable with less than six parents many of the 'trial' parenting groups included families who were not part of the trial. We failed to anticipate this and as a result we have had to film and will need to code many more sessions than we originally expected.

A further challenge to studies of interventions which often involve complex changes in behaviour or family relationships is to choose an appropriate outcome measure. Well validated measures are essential for the credibility of the results yet such measures may not capture all possible changes that may occur. For this reason, we have opted to collect multiple outcome measures both self report and objective, but this has increased respondent burden and also researcher time and therefore the cost of the trial. The primary outcome is a composite measure which has worked well in other studies, we have examined the properties of this measure in the baseline data to ensure that it is statistically sound in the current study and presented the findings to the Trial Steering Committee for confirmation of the primary outcome before completion of data collection.

Gathering video data is valuable in providing objective evidence. In this trial it proved challenging for some parents who found it difficult to trust the research team to maintain confidentiality. It also proved challenging to facilitators who needed to be videoed for fidelity coding. It is easy to underestimate how nervous skilled group facilitators may be in the face of possible when a judgement of their skills.

Hutchings *et al *reflect on lessons learnt from running three pragmatic randomised controlled trials of child mental health interventions in Wales [53], six themes emerge;

1. Identification of suitable partner services for trials

2. Early recruitment of service managers in study planning and ongoing shared management

3. Clarification of contributions/requirements from all partners

4. Ethical and sensitive recruitment of participants

5. Building an understanding of research evaluation

6. Enhancing the fidelity of the programme [53]

We concur with these insights. This has been an extremely challenging trial to run. It could have failed at several points, but is now on course to succeed. The key to success has been an absolutely committed, very hard working team of researchers and experienced academics across two universities, an absolutely committed supportive group of early years commissioners and managers in South Wales and the hard work of a great many practitioners working on the ground to recruit families.

## Abbreviations

USA: United States of America; UK: United Kingdom; FLNP: Family Links Nurturing Programme; RCT: Randomised Controlled Trial; IY: Incredible Years; TP: Triple P; HOME inventory: Home Observation and Measurement of the Environment Inventory; MORS: Mothers' Object Relations Scales; PedsQL: Pediatric Quality of Life; prePACS: pre-Parent Account of Child Symptoms; WEMWBS: Warwick-Edinburgh Mental Well-Being Scale; SF-6D: Short Form 6 Dimension; CRCC: Clinical Research Collaboration Cymru.

## Competing interests

The authors declare that they have no competing interests.

## Authors' Contributions

DS helped to conceive and design the study and drafted the manuscript. HS helped to conceive and design the study and critically revised the manuscript for intellectual content. NS participated in the design of the study and will perform the statistical analysis. SD helped to design the study and performed the economic analyses. MT, BA, SW and LW contributed to the implementation and discussion sections of the manuscript. SSB conceived of the study and participated in its design and coordination and helped to draft the manuscript. All authors have read and approved the final manuscript.

## Pre-publication history

The pre-publication history for this paper can be accessed here:

http://www.biomedcentral.com/1471-2458/10/364/prepub
